# A microRNA activity map of human mesenchymal tumors: connections to oncogenic pathways; an integrative transcriptomic study

**DOI:** 10.1186/1471-2164-13-332

**Published:** 2012-07-23

**Authors:** Elena Fountzilas, Andrew D Kelly, Antonio R Perez-Atayde, Jeffrey Goldsmith, Panagiotis A Konstantinopoulos, Nancy Francoeur, Mick Correll, Renee Rubio, Lan Hu, Mark C Gebhardt, John Quackenbush, Dimitrios Spentzos

**Affiliations:** 1 Division of Hematology/Oncology, Sarcoma Program, Department of Medicine, Beth Israel Deaconess Medical Center, Harvard Medical School, 330 Brookline Avenue, Boston, MA, 02215, USA; 2 Department of Pathology, Beth Israel Deaconess Medical Center, Harvard Medical School, Boston, MA, 02215, USA; 3 Center for Cancer Computational Biology, Department of Biostatistics and Computational Biology, Dana-Farber Cancer Institute, Boston, MA, 02215, USA; 4 Department of Pathology, Boston Children's Hospital, Harvard Medical School, Boston, MA, 02215, USA; 5 Department of Orthopedic Surgery, Beth Israel Deaconess Medical Center, Harvard Medical School, Boston, MA, 02215, USA

**Keywords:** MicroRNA, Microarray, RAS, Mesenchymal tumors, MicroRNA biogenesis

## Abstract

**Background:**

MicroRNAs (miRNAs) are nucleic acid regulators of many human mRNAs, and are associated with many tumorigenic processes. miRNA expression levels have been used in profiling studies, but some evidence suggests that expression levels do not fully capture miRNA regulatory activity. In this study we integrate multiple gene expression datasets to determine miRNA activity patterns associated with cancer phenotypes and oncogenic pathways in mesenchymal tumors – a very heterogeneous class of malignancies.

**Results:**

Using a computational method, we identified differentially activated miRNAs between 77 normal tissue specimens and 135 sarcomas and we validated many of these findings with microarray interrogation of an independent, paraffin-based cohort of 18 tumors. We also showed that miRNA activity is imperfectly correlated with miRNA expression levels. Using next-generation miRNA sequencing we identified potential base sequence alterations which may explain differential activity. We then analyzed miRNA activity changes related to the RAS-pathway and found 21 miRNAs that switch from silenced to activated status in parallel with RAS activation. Importantly, nearly half of these 21 miRNAs were predicted to regulate integral parts of the miRNA processing machinery, and our gene expression analysis revealed significant reductions of these transcripts in RAS-active tumors. These results suggest an association between RAS signaling and miRNA processing in which miRNAs may attenuate their own biogenesis.

**Conclusions:**

Our study represents the first gene expression-based investigation of miRNA regulatory activity in human sarcomas, and our findings indicate that miRNA activity patterns derived from integrated transcriptomic data are reproducible and biologically informative in cancer. We identified an association between RAS signaling and miRNA processing, and demonstrated sequence alterations as plausible causes for differential miRNA activity. Finally, our study highlights the value of systems level integrative miRNA/mRNA assessment with high-throughput genomic data, and the applicability of paraffin-tissue-derived RNA for validation of novel findings.

## Background

Early research on microRNAs (miRNAs) has demonstrated their critical function in a variety of neoplastic processes and has further highlighted the molecular complexity of cancer [1-5]. One of the most complex and heterogeneous cancer types is the group of malignant mesenchymal tumors (also known as sarcomas). There are few reliable biomarkers for sarcoma classification and the molecular underpinning of their heterogeneous behavior remains poorly understood [6,7]. Early work has shown that miRNA expression levels can be used to distinguish between sarcoma subtypes [8]. However, expression levels do not necessarily signify activity in terms of effects on their target mRNAs; there is evidence that miRNA activity can be increased irrespectively of miRNA expression levels [9,10]. Given the increasing amount of gene expression data now available in the public domain, the concept of inferring miRNA activity using gene expression profiles as a surrogate has been proposed [9,11]. This method combines miRNA target predictions based on sequence complementarity with concerted changes in the expression levels of corresponding target mRNAs [11]. Thus, the output is an inferred level of miRNA regulatory activity. In this study we sought to identify miRNA activity patterns in sarcomas by integrating gene expression data from multiple sources and using a recently developed computational algorithm [11]. On this basis, miRNAs were defined as either activated or silenced in tumors (not necessarily equivalent to over or under-expressed). We then validated potentially altered miRNAs by profiling an independent paraffin-derived sarcoma cohort and investigating their possible connection with oncogenic pathway activity. We also performed RNA-sequencing to identify possible miRNA sequence alterations and we propose a link between the RAS pathway and mature miRNA biogenesis.

## Methods

### Gene expression datasets

We used four public datasets, (oligonucleotide Affymetrix U133A), from Japan [12], Memorial Sloan Kettering Cancer Center (MSKCC) [13], UK [14] and Genomics Institute of the Novartis Research Foundation (GINRF) [15]. Raw data were retrieved for a total of 77 normal tissue samples, including epithelial/adenoid (44), hematopoietic (1), neuroendocrine (6), gonadal (4), neural (9) and mesenchymal tissues (13), and 135 sarcoma samples (including 28 non-myxoid liposarcomas comprised of 6 well-differentiated, 3 pleomorphic, and 19 dedifferentiated, 30 round cell/myxoid liposarcomas, 16 fibrosarcomas, 30 synovial sarcomas, 20 leiomyosarcomas, and 11 osteosarcomas – available in only one dataset). The data were processed using the Robust Multi-Array Average (RMA) algorithm. Non-biological experimental variation (batch effect) between the datasets was corrected using a previously described algorithm [16]. The compendium of these public datasets was used as a discovery set to identify candidate miRNAs with deregulated activity.

For comparison purposes we processed raw data in a similar manner from non-sarcoma datasets. Specifically, we used three publicly available ovarian cancer (Duke [17], Michigan [18], UPenn [19]) and three head and neck cancer datasets (UPenn [20], University of Medicine and Dentistry of New Jersey [21], UWisconsin [22]), all oligonoucleotide Affymetrix U133A or U133 2.0 plus.

### Paraffin-based validation cohort

We used 18 formalin-fixed paraffin-embedded (FFPE) sarcoma samples from the pathology archive of Beth Israel Deaconess Medical Center (BIDMC) and Boston Children's Hospital (BCH). This work was done in accordance with a protocol for archival tissue collection and use which was approved by the Institutional Review Board (IRB) at both institutions. The requirement for a patient consent form was waived by the IRB at BIDMC. This cohort included 4 liposarcomas (all well-differentiated, non-myxoid), 3 leiomyosarcomas, 2 synovial sarcomas, and 9 osteosarcomas.

### FFPE RNA isolation, whole genome and miRNA profiling

FFPE samples were cut into 1–3 mm cores. Total RNA was isolated using the Qiagen RNeasy FFPE protocol. Whole genome c-DNA-mediated annealing, selection, extension, and ligation (DASL) arrays, (Illumina, CA) containing probes for 24,000 annotated genes, were used for profiling. The DASL assay is a bead-based method for expression profiling of degraded RNA, such as that extracted from FFPE samples [23-27]. Similarly, miRNA expression profiling was performed using miRNA DASL assays, containing probes for 1146 miRNAs [28,29]. Raw miRNA and mRNA DASL data have been deposited in NCBI’s Gene Expression Omnibus (GSE35851, and GSE35852) [30].

The expression profiling experiments were performed at the Molecular Genetics Core at BCH. Normalization was performed following manufacturer instructions (Genome Studio^TM^, Gene Expression Module v1.0 User Guide, Illumina). Background subtracted sample intensities were scaled by a factor equal to the ratio of average intensity of a virtual reference sample to the average intensity of a given sample.

### Small RNA sequencing

Total RNA samples were prepared for smRNA sequencing using Illumina’s Small RNA v1.5 Sample Preparation Guide. Total RNA input ranged from 5-10 μg and first underwent 3′ and 5′ adaptor ligation followed by reverse transcription and 12 cycles of amplification on a Bio-Rad iCycler. cDNA constructs were then purified using a 6% Novex TBE PAGE gel on Invitrogen’s XCell SureLock Novex Mini-Cell System. Band sizes ranging from 80-100 bp were cut from the gel and purified. cDNA constructs were eluted from the gel and purified by ethanol precipitation according to Illumina’s protocol. Libraries were analyzed on Agilent’s 2100 Bioanalyzer with a High Sensitivity DNA Chip specific for next generation sequencing. Final libraries were immobilized onto a single read Illumina flowcell at a concentration of 12pM and underwent cluster amplification on Illumina’s Cluster Station using their DGE Small RNA Cluster Generation Kit. The amplified flowcell was then sequenced on Illumina’s GAIIx with 36 cycles of sequencing.

### miRNA read mapping and quantification

The leading 21 bases were trimmed from the 36-bp reads based on the quality score and the length of mature miRNAs. The trimmed reads were mapped to miRNA precursor sequences in miRBase 16.0 [31] to achieve more sensitive expression profiles using the software miRExpress [32]. One base difference between the reads and the miRNA precursor sequences was allowed, which covered exact match, one gap, one base insertion, and one base difference. The number of reads mapped to a miRNA sequence was taken to represent the expression level of that miRNA.

### miRNA activity algorithm

To assess miRNA activity patterns we used a recently described algorithm [11] designed to take a set of gene expression changes as a surrogate to determine relative miRNA activity across two conditions. The algorithm is based on the premise that expression changes of the target genes (miRanda target prediction algorithm) of a certain miRNA between two conditions reflect its activity. In brief, the expression changes are ranked in a decreasing order (expression change vector). Next, the expression change vector is screened for the distribution of genes with high binding affinity for a certain miRNA. Under the null hypothesis of no miRNA activity change, genes with high and low binding affinities will position randomly in the expression change vector. Thus, miRNA activity (or silencing) inference can be made if the distribution of gene targets for a specific miRNA is skewed on the expression vector. A positive activity score (AS) indicates the miRNA has inferred activation, while a negative activity score indicates miRNA silencing.

### Estimation of false discovery rate

An estimated false discovery rate (FDR) was based on permutations of the gene expression data as previously described [11]. In brief, for each miRNA (x) activity scores are calculated for the original data (AS(x)), and also for each of 1000 random permutations (k) of the gene labels in the mRNA expression data (NS(x, k)). NS(x, k) for all x and k is then used as the null distribution for FDR calculation for a given AS(x) = AS*. If AS* ≥ 0, the FDR estimate for miRNA x* is then defined as the ratio of the percentage of all (x, k) where NS(x, k) ≥ 0, and NS(x, k) ≥ AS*, divided by the percentage of miRNAs with AS(x) ≥ 0, where AS(x) ≥ AS*, and similarly if AS* < 0 [11].

### Functional representational analysis

To explore biological themes in the miRNA activity patterns we used functional representational analysis, as previously described [33]. For each biologic theme, an EASE (Expression Analysis Systematic Explorer) score is calculated based on the over-representation, or lack thereof, of genes belonging to that theme in the gene pattern discriminating two conditions. The EASE score is an adjusted Fisher’s test, further modified by the FDR method.

### Hierarchical clustering

Clustering was performed using the average linkage method implemented in the NCI BRB Array Tools software [34,35].

### Predictions of RAS activation

We retrieved gene expression “read outs” of RAS activation previously validated by controlled RAS activation *in vitro.* These “read outs” were used to train Bayesian probit regression models of pathway activity [36]. We applied these models to assign a probability of pathway activation in individual sarcoma samples in our study. Non-biological experimental variation between datasets was corrected using the batch effect adjustment algorithm as above. In order to afford high confidence for activity calls a probability of 0.8 was the minimum for predicted pathway activation.

### Assessment of RAS-associated miRNA targets

The predicted mRNA targets of “RAS-switching” miRNAs were identified using the TargetScan and miRanda algorithms (both available online) [37,38]. Relevant transcript levels between RAS-active and RAS-inactive tumors were compared using a 1-tailed *t*-test assuming heteroskedasticity.

## Results

### miRNA activity in the different sarcoma histologies

**Figure 1:**
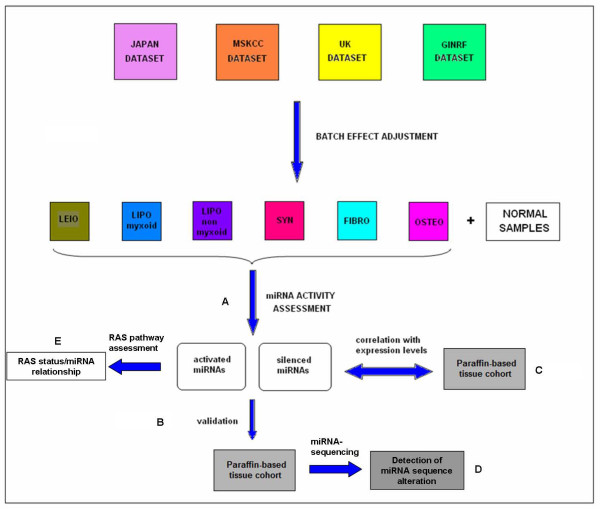
**Study flow.A**) miRNA activity pattern assessment in four public datasets. **B**) Validation in a paraffin-based tissue cohort. **C**) Correlation of miRNA activity with miRNA levels. **D**) miRNA-sequencing. **E**) Relationship with RAS pathway status.

The workflow of our study is described in Figure 1. We integrated sarcoma and normal tissue samples from the four public datasets and we adjusted for non-biological experimental variation. This adjustment is important when attempting integrated analysis of multiple microarray datasets to eliminate results reflecting non-biological technical variation between datasets. We performed the analysis separately for each histology (leiomyosarcoma - LEIO, myxoid liposarcoma - LIPO myxoid, non-myxoid liposarcoma - LIPO non-myxoid, synovial sarcoma - SYN, fibrosarcoma - FIBRO) compared to the normal tissue arrays as the comparator phenotype. We observed a set of activated or silenced miRNAs in all sarcoma histological subtypes compared to normal tissue samples (Table 1, Additional file 1: Table S1 p = 0.005 and FDR = 0.01). Most of these miRNAs were commonly identified as differentially activated in all sarcoma subtypes compared to normal tissue samples (all Fisher’s exact test p < 2e-16), suggesting that they may reflect generic changes related to cancer transformation. There was also a subset of non-overlapping miRNAs (Table 2) which may be more specific to the different sarcoma differentiation lines. We reasoned that we might gain further insight into the specific sarcoma miRNA activity patterns by limiting the comparator phenotype to the normal mesenchymal tissue and the results of this analysis are shown in Table 1 and Additional file 1: Table S1. Using this procedure, we also identified 18 miRNAs with a unique sarcoma subtype-specific activity pattern with respect to normal mesenchymal tissue (Table 2). Several of these miRNAs were also identified as differentially activated with respect to the initial normal tissue comparator and are denoted in Table 2. We also explored miRNA activity in osteosarcoma (OSTEO) samples. Comparing the deregulated miRNAs from this analysis with the respective miRNAs from the soft-tissue sarcoma analysis we identified 12 miRNAs with unique activity in osteosarcoma (Table 2, Additional file 1: Table S2, Table S3).

**Table 1:** miRNA activity patterns in sarcoma subtypes

	**VS ALL NORMAL TISSUE**		**VS MESENCHYMAL NORMAL TISSUE**	
**HISTOLOGICAL SUBTYPE**	**Activated miRNAs**	**Silenced miRNAs**	**Activated miRNAs**	**Silenced miRNAs**
**LEIO**	67	27	71	41
**myxoid LIPO**	65	27	59	46
**Non-myxoid LIPO**	69	25	60	54
**FIBRO**	69	33	66	53
**SYN**	62	28	53	63
**COMMON in all histologies**	**59**	**17**	**52**	**35**

Shared miRNA activity profiles among the different histological subtypes compared to all normal tissue and to mesenchymal normal tissue (p = 0.005 and FDR = 0.01).

**Table 2:** Histology-specific miRNA deregulation patterns

**LEIO**	**LIPOm**	**FIBRO**	**SYN**	
**hsa-miR-128b**				
***hsa-miR-100***				
***hsa-miR-99a***	hsa-miR-17-3p¹	hsa-miR-107¹	**-**	**Activated**
***hsa-miR-212***		hsa-miR-128a		**miRNAs**
**hsa-miR-199b**				
**hsa-miR-98¹**				
			hsa-miR-217¹	
			hsa-miR-181a	
			**hsa-miR-330**	
		hsa-miR-30a-3p	**hsa-miR-29c**	
hsa-miR-374	hsa-miR-302a*	hsa-miR-154*	**hsa-miR-221**	**Silenced miRNAs**
**hsa-miR-361**	hsa-miR-130	hsa-miR-21¹	hsa-miR-217¹	
		**hsa-miR-208**	**hsa-miR-424**	
			**hsa-miR-25**	
			hsa-miR-126*	
			**hsa-miR-31**	
			**hsa-miR-302a**	
			**hsa-miR-26a¹**	
**OSTEOSARCOMA SPECIFIC miRNAs**				
***hsa-miR-122a***	hsa-miR-7 g			
hsa-miR-18	***hsa-miR-147***			
***hsa-miR-34c***	***hsa-miR-210***	**Activated mIRNAs**		
**hsa-miR-375**	***hsa-miR-187***			
***hsa-miR-204***	***hsa-miR-134***			
***hsa-miR-138***	***hsa-miR-211***			

Boldface denotes activated or silenced miRNAs compared to mesenchymal normal tissue only. Boldface italicized text denotes activated or silenced miRNAs compared to both mesenchymal normal tissue and all normal tissue. A superscript 1 denotes miRNAs which are also differentially activated in epithelial cancers.

### Validation of miRNA activity patterns in a paraffin tissue cohort

**Figure 2:**
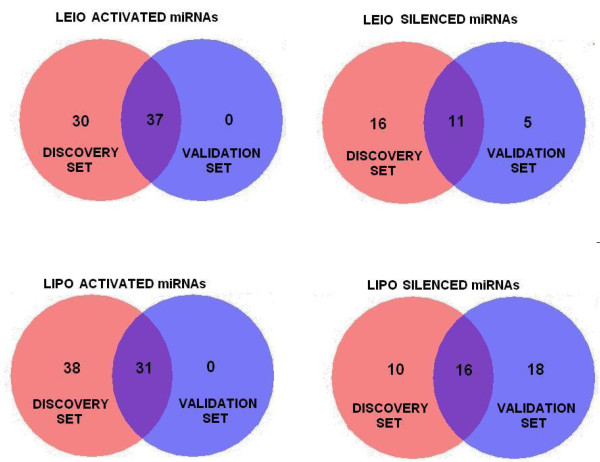
**Validation of miRNA activity patterns in a paraffin-based tissue cohort.** Activity patterns of many dysregulated candidate miRNAs were reproducible in the validation set (LEIO and LIPO samples, p = 0.005, FDR = 0.05).

To validate the results obtained from the integrated gene expression dataset we used an FFPE sarcoma tissue cohort previously profiled by our group using DASL [39]. We analyzed miRNA activity for LEIOs and LIPOs, which were the most abundant subtypes represented in that dataset (3 LIPOs, 3 LEIOs). Despite the relatively small number of FFPE samples, a large fraction of the candidate miRNAs was again found to be deregulated with respect to all normal tissue specimens exactly as predicted by the discovery set (Figure 2, p = 0.005 and FDR = 0.05, all Fisher’s exact test p < 4e-8). When we used only the mesenchymal tissue subset as the comparator in the validation cohort, the overlap was also very high (all Fisher’s exact test p < 0.0016). For the leiomyosarcomas in the validation set, 25 miRNAs were found to be activated and 12 were silenced. All except one of these miRNAs was respectively identified as activated or silenced in the discovery set (25/25, 11/12). For the liposarcomas, 5 miRNAs were found to be activated and 23 were silenced. All 5 of the activated miRNAs were also activated in the discovery set, and 21 of the silenced miRNAs were also silenced in the discovery set. Thus, the reproducibility was unlikely to be limited by type of normal tissue comparator.

### Differentially active miRNAs may harbor a sequence alteration

To investigate possible etiologies for differential activity, we performed miRNA-sequencing on one leiomyosarcoma, and one non-myxoid liposarcoma from the validation set with the hypothesis that miRNA sequence alterations may account – at least in part – for activity changes. The samples were each run in technical duplicate on the Illumina GAIIx platform. By comparing exact sequence mapping to reference miRNAs with sequence mapping allowing for a single base difference between reads and the reference, we identified several differentially activated miRNAs with potential single base alterations in both technical replicates for both samples (Tables 3 and 4). As an example, sequencing reads for the differentially activated miRNA, miR-422a are shown in Table 3 with a complete list of miRNAs in Table 4. We observed that in addition to reads mapping directly to reference miRNA sequences, there were also a substantial number of reads (distinct from the reference by one base) which mapped to no region of the human genome, suggesting either post-transcriptional modification, or copy number changes combined with mutation. While a potential limitation of our results would be if there is an unknown sequence-specific bias in our platform or if we are detecting novel miRNAs, we are fairly confident that miRNA alterations exist in these tissue samples. Because the sequencing read length (36 bases) is longer than the length of the mature form of miRNAs, and because two independent samples which underwent independent sequencing library preparation were run in duplicate on four flow cell lanes, there is little chance that experimental variability could account for all of the possible alterations described. This is further supported by base call quality scores from the FastQC report which imply an estimated base call accuracy of 99.9% (mean score 30).

**Table 3:** Example of a potential miRNA sequence alteration

**hsa-miR-422a ACUGGACUUAGGGUCAGAAGGC**			
**Sample**	**Sequencing Reads**	**Counts (≤1 Bases Different)**	**Counts (0 Bases Different)**
LEIO_1	ACUGGACUU - GGGUCAGAAGGC	10	0
LEIO_2	ACUGGACUU - GGGUCAGAAGGC	15	0
LIPO_1	ACUGGACUU - GGGUCAGAAGGC	25	0
LIPO_2	ACUGGACUU - GGGUCAGAAGGC	15	0

The reference mature sequence of miR-422a is shown along with RNA-sequencing reads for each duplicate of one leiomyosarcoma (LEIO) and one liposarcoma (LIPO). The column denoted by " ≤ 1 bases different" reports the number of sequencing reads when allowing for a single base difference in mapping to the reference. The column denoted by "0 bases different" reports sequencing reads when allowing for no differences in mapping to the reference.

**Table 4:** Differentially activated miRNAs with possible sequence alterations

**VS ALL NORMAL TISSUE SAMPLES**		**VS MESENCHYMAL NORMAL TISSUE SAMPLES**	
**Activated miRNAs**	**Silenced miRNAs**	**Activated miRNAs**	**Silenced miRNAs**
hsa-let-7e	hsa-miR-186	hsa-miR-328	hsa-miR-19a
hsa-miR-24	hsa-miR-19b	hsa-miR-324-5p	hsa-miR-19b
hsa-miR-185	hsa-miR-101	hsa-miR-24	hsa-miR-186
hsa-let-7c	hsa-miR-203	hsa-miR-378	hsa-miR-32
hsa-let-7i	hsa-miR-200b	hsa-let-7b	hsa-miR-203
hsa-miR-22	hsa-miR-32	hsa-miR-125b	hsa-miR-26b
hsa-miR-125b	hsa-miR-19a	hsa-let-7c	hsa-miR-200b
hsa-miR-378	hsa-miR-26b	hsa-miR-340	hsa-miR-101
hsa-let-7d		hsa-miR-214	
hsa-miR-197		hsa-let-7e	
hsa-miR-214		hsa-miR-34a	
hsa-miR-340		hsa-let-7d	
hsa-miR-34a		hsa-let-7i	
hsa-let-7b		hsa-miR-422a	
hsa-miR-145		hsa-let-7a	
hsa-miR-324-5p		hsa-miR-197	
hsa-miR-328		hsa-miR-425	
hsa-miR-210		hsa-miR-185	
hsa-miR-425		hsa-miR-210	
hsa-miR-422a		hsa-miR-145	
hsa-let-7a		hsa-miR-22	

A subset of differentially activated miRNAs with respect to all normal tissues (left columns) or mesenchymal normal tissues (right columns), that harbor possible sequence alterations.

### Imperfect correlation between miRNA activity and miRNA expression levels

**Figure 3:**
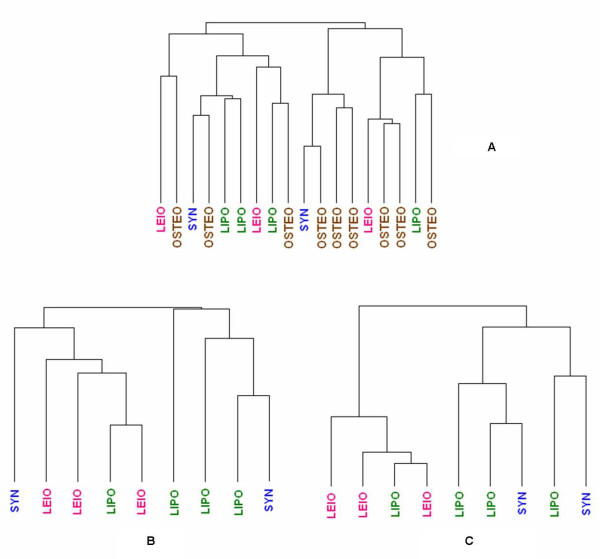
**Imperfect correlation between miRNA activity and miRNA expression levels.** Hierarchical clustering based on histology-specific miRNAs: **A**) Using all samples (soft-tissue sarcomas and osteosarcomas), **B**) using only soft-tissue sarcomas, **C**) using soft-tissue sarcomas while limiting the analysis to the most variant miRNAs.

Based on recent observations, the intuitive question – which is also highlighted by the finding of potential miRNA sequence alterations above – of whether miRNA expression levels correlate well with miRNA activity in human tissue has been raised, and we have explored this for the first time in sarcoma [40]. Because the public sarcoma datasets used lacked miRNA expression data and our previously profiled paraffin dataset lacked normal tissue samples, we could not directly compare miRNA activity changes and expression levels in either the public frozen tissue-based or the paraffin-based datasets, therefore, we used an indirect approach. We performed supervised hierarchical clustering using the expression *levels* of the sarcoma subtype-specific miRNAs, (chosen based on *activity* in the discovery set) and observed whether the FFPE sarcoma samples would separate based on histology. Our analysis demonstrates that they did not (Figure 3). Given the possibility of confounding by inclusion of osteosarcomas, we attempted to cluster the samples excluding the osteosarcomas and again we did not observe a reasonable separation. Finally we limited our analysis to the top 50% most variant miRNAs (in terms of expression) and observed an improvement on the separation of the soft-tissue sarcoma samples. These results suggest that miRNA activity is not perfectly correlated with miRNA expression levels although the correlation might be stronger with larger expression changes.

### Sarcomas demonstrate partially different miRNA activity patterns compared to epithelial cancers

To investigate the degree to which miRNA activity patterns that we discovered are unique to sarcoma, we compared samples from three ovarian and three head and neck cancer datasets with the same normal tissue samples (from GINRF) that we had previously used for the sarcoma analysis, and identified differentially activated miRNAs. This analysis revealed that the majority of the histology specific miRNAs described above were unique to sarcoma and were not shared with the epithelial tumors (23/28 miRNAs were unique to sarcoma; Table 2).

However, we also found that the miRNAs which were commonly activated in sarcomas with respect to both all normal tissue and mesenchymal normal tissue highly overlapped (50 out of 53 miRNAs; Fisher’s exact test p < 2e-45) with the miRNAs which were commonly activated in both the HNC and Ovarian cancer tissue samples. Interestingly, the same was not true of commonly silenced miRNAs in the sarcoma subtypes. Of the 17 miRNAs commonly silenced in sarcomas with respect to both all normal and mesenchymal tissue, only 1 was also commonly silenced in both the HNC and Ovarian samples (Additional file 1: Table S4). Therefore, it appears that many activated miRNAs are common to epithelial cancers, and may represent a more general cancer phenomenon. There are, however, several silenced miRNAs which are common to all sarcoma histological subtypes which appear to be silenced only in sarcomas.

### RAS pathway status is associated with miRNA activity and mature miRNA biogenesis

In order to further explore possible biological connections with important cancer pathways, we hypothesized that sarcoma phenotypes characterized by distinct activation of a known oncogenic pathway may demonstrate different miRNA activity patterns. In order to test this, we compared miRNA activity patterns between the sarcoma samples that demonstrated RAS pathway activation to those that did not. The pathway activation predictions were made based on published gene expression signatures of oncogenic pathway activation [36]. There was some variation in the prevalence of RAS activity across histological subtypes. The fractions of “RAS active” samples were 19/28, 6/30, 9/16, 3/30, and 8/20 for non-myxoid liposarcoma, myxoid liposarcoma, fibrosarcoma, synovial sarcoma, and leiomyosarcoma respectively. Indeed, we found that both in aggregate (all subtypes taken together) and in a subtype-specific manner, samples separated by RAS activity status demonstrated different activity profiles. Specifically, we identified 42 miRNAs activated in the aggregate “RAS active” group and 30 miRNAs silenced in aggregate “RAS non-active” group (Additional file 1: Table S5, Table S6; p = 0.005 and FDR = 0.01). Among these miRNAs, 21 were present in both lists (Fisher’s exact test p < 6e-11), suggesting that these miRNAs may reverse their activity upon transition to RAS-active tumor status (“RAS-switching” miRNAs). Furthermore, it has been shown that miR-7 – one member of this list – is transcribed as a result of RAS signaling [41]. When we examine RAS-associated miRNA activity changes by specific subtype, the results for non-myxoid liposarcomas and synovial sarcomas are largely overlapping with the aggregated analysis, however, it does not appear that there are significant RAS-switching miRNAs in the other histological subtypes (Additional file 1: Table S7).

Interestingly, many of the RAS-switching miRNAs from the aggregate, non-myxoid liposarcoma, and synovial sarcoma analyses have predicted mRNA targets which translate to proteins in the miRNA processing machinery. Using both TargetScan and miRanda, we found that six of the twenty-one miRNAs from the aggregate analysis are predicted to target AGO2, four are predicted to target DROSHA, four target DICER1, three target TRBP, and one targets DGCR8. In all, nine of the twenty-one identified RAS-switching miRNAs target one or more of the established miRNA processing genes. Furthermore, miR-144, identified as switching specifically in non-myxoid liposarcoma, and synovial sarcoma, is predicted to target DICER1. A summary of these findings is presented in Table 5. To evidence that these miRNAs actually target the processing machinery genes, we examined the transcript levels of these genes (determined by microarray) in RAS-active tumors relative to RAS-inactive tumors, with the hypothesis that they would be down-regulated. Indeed, we observed statistically significant down-regulation of TARBP2, DICER1, DROSHA, and DGCR8 in RAS-active tumors (1-tailed t-tests: p = 0.00056, 0.0019, 1.24e-5, and 0.00020 respectively). This indicates that RAS status may be related to a miRNA-based regulation of global miRNA processing.

**Table 5:** Summary of predicted RAS-related miRNA targets

**miRNA**	**RAS + Tumors**	**RAS- Tumors**	**Target Scan Predictions**	**miRanda Predictions**
hsa-miR-200b	On	Off		DROSHA
hsa-miR-27b	On	Off	EIF2C2, DROSHA	EIF2C2, DROSHA
hsa-miR-424	On	Off	DICER1, TARBP2	EIF2C2, TARBP2
hsa-miR-99a	On	Off	EIF2C2	EIF2C2
hsa-miR-200c	On	Off		DROSHA
hsa-miR-31	On	Off	DICER1, DGCR8	DICER1, DGCR8
hsa-miR-15a	On	Off	DICER1, TARBP2	EIF2C2, DICER1, TARBP2
hsa-miR-16	On	Off	DICER1, TARBP2	EIF2C2, DICER1, TARBP2
hsa-miR-27a	On	Off	EIF2C2, DROSHA	EIF2C2, DROSHA

Predicted mRNA targets of "RAS-switching" miRNAs related to miRNA processing are summarized. The columns denoted "RAS + Tumors" and "RAS- Tumors" indicate whether RAS-switching miRNAs are activated (On) or inactivated (Off) relative to normal tissue for RAS-active and RAS-inactive tumors respectively.

### Biological themes represented in distinct miRNA activity patterns

To identify other possible biological mechanisms that may be perturbed by miRNA activity changes we used predicted gene targets for each histology-specific miRNA to discover biological themes overrepresented in these target gene sets. We identified a number of biological themes that seem to be shared by the majority of the sarcoma subtypes. However, there were some unique themes in each histological subtype, for instance the extracellular matrix and inflammatory response pathways in synovial sarcoma. The full list of biological themes is presented in Additional file 1: Table S8 (EASE Score = 0.05, global FDR = 0).

## Discussion

miRNAs have been shown to play a critical role in many biological processes, including cell proliferation, cell cycle, differentiation and apoptosis [1-5]. Their primary function was initially thought to be the direct inhibition of translation, but they are now recognized to target mRNAs for degradation [42]. It has been suggested that the effect of a miRNA on its target mRNA depends on the strength of their binding and the degree of sequence complementarity. Under this paradigm, perfect pairing leads to mRNA degradation, while imperfect pairing results in translation inhibition [43]. Until recently, most miRNA studies have focused on expression levels, but clinical data on miRNA activity are lacking and it is unclear if miRNA expression levels are a good surrogate for activity.

Sarcomas – a uniquely complex group of mesenchymal tumors – are perfect candidates for exploring the regulatory role of miRNAs with the aims of better understanding their biology, and developing clinical biomarkers and therapeutic targets. To our knowledge, there is limited information on the role of miRNAs in sarcoma. Subramanian *et al.* used miRNA expression levels to characterize various sarcoma subtypes with distinct miRNA profiles, thereby supporting the possible importance of miRNAs in the biology of these tumors [8].

Our goal was to determine miRNA activity in some of the most common sarcoma subtypes with a recently developed algorithm which uses sarcoma gene expression data as a surrogate. We identified several miRNAs that appear specifically deregulated in each sarcoma subtype, using normal tissue as a comparator. Despite the technical challenges associated with confirming *in silico* findings, we validated the deregulated activity of many of these candidate miRNAs using a paraffin-based cohort. The majority of these miRNAs were shared in all histological subtypes, suggesting that they are perhaps related to a general neoplastic transformation. Another subset, however, appeared to be unique to each sarcoma subtype. In order to further corroborate the miRNA specificity for each sarcoma subtype, we performed miRNA activity analysis using ovarian and head and neck cancer datasets and the same normal tissue cohort as a comparator. This analysis demonstrated that the majority of the sarcoma subtype-specific miRNAs were also truly unique to sarcoma subtypes. At the same time, our findings support the notion that certain common miRNA activity changes in sarcomas may be related to a general cancer phenotype as nearly all of these miRNAs were also activated in both the ovarian and head and neck tumors.

We were then interested in uncovering potential etiologies for differential activity, one example being mature miRNA sequence alterations. Using RNA-sequencing on two of the FFPE specimens from our validation cohort we found that several miRNAs which we identified as differentially activated in all sarcomas relative to normal tissue harbor possible sequence alterations. Whether this is indicative of mutation or post-transcriptional processing is unclear because we did not perform genomic DNA sequencing, but nevertheless, an impact on miRNA activity could be explained by either phenomenon. We reason that a miRNA base deletion could conceivably lead to either increased or decreased activity because target complementarity may be either increased or decreased as a result. Another explanation for differential activity could be the presence of a chromosomal translocation. We identified the chromosomal locations of miRNAs identified as sarcoma subtype-specific in our study, and we found that miR-221, which was uniquely silenced in synovial sarcoma in our analysis, is located at Xp11.3, very near the common synovial chromosomal translocation t(X;18)(p11.2, q11.2) [44]. Rigorously investigating all possible reasons for differential activity is beyond the scope of this study, but our findings regarding potential miRNA sequence alterations suggest that mutation, post-transcriptional modification, and/or chromosomal aberrations may play a prominent role.

To explore how differential miRNA activity may manifest characteristic phenotypic states in cancer, we evaluated the relationship between miRNA activation and RAS signaling. We categorized sarcoma samples as RAS-active versus RAS-inactive using previously validated expression “read outs” of RAS activity [36]. The data demonstrated that, in aggregate, sarcomas with active RAS were characterized by different miRNA activity profiles compared to sarcomas without active RAS and, interestingly, a subset of miRNAs appeared to “switch” activity between the two pathway “classes.” We also examined the distributions of RAS status with respect to histological subtype and found considerable variability in the rates of RAS activation. This suggests that RAS pathway activity may be sarcoma-subtype-specific *per se*. Performing the activity analysis separately on each of the histological subtypes revealed that significant “RAS-switching” miRNAs were present in only the non-myxoid liposarcomas and the synovial sarcomas. Interestingly, one of these miRNAs, miR-7, has been shown to promote tumorigenesis via regulation by a mechanism in which RAS signaling increases miR-7 transcription [41]. We propose that the increased expression of miR-7 in some RAS-active sarcomas also leads to increased miRNA activity as determined by our computational approach. A very interesting finding is that many of the identified RAS-switching miRNAs have predicted mRNA targets which encode proteins in the endogenous miRNA processing machinery. In all, nearly half of these miRNAs target one or more of the processing protein transcripts, and we confirmed significantly decreased expression of these mRNAs in RAS-active tumors. We therefore hypothesize that miRNA repression of processing proteins contributes to the observed down-regulation of some miRNAs in human tumors. This seems plausible as a similar phenomenon of Dicer regulation has been described [45]. These observations require further work to examine whether the miRNA activity changes are contributory or causal in the RAS activation process, and to examine the link between miRNA processing machinery, RAS, and miRNA activity.

In addition to exploring miRNA activity, our study addresses the question of whether miRNA expression levels are reasonable surrogates for activity in sarcomas. Our data suggest that there is an imperfect relationship between activity and expression levels, and that it may be stronger for highly variant (in terms of expression level) miRNAs. It has been suggested that dramatic changes in miRNA levels may predictably result in activity changes, but activity can change even with small changes in expression level for various other reasons [46,47]. For instance, functional alterations of proteins that have a role in the RNA-induced silencing complex (RISC), such as Argonaute, can cause activity changes without affecting miRNA levels [46]. miRNA mutations can also cause altered miRNA activity while leaving the miRNA expression levels measured by microarray intact. Finally, it has been shown that certain transcripts may act as miRNA “sponges,” whereby miRNA regulatory effects may be modulated without changing their expression levels [47]. Supporting these notions is a comparison of our findings and those of Subramanian *et al.* based on expression levels [8]. We found only two synovial sarcoma-specific miRNAs, miR-126 and miR-129, that have both lower expression levels and decreased activity in both studies. While this question merits further study, these observations support the notion that expression levels and target mRNA levels capture different aspects of miRNA regulatory activity in sarcomas.

## Conclusions

In conclusion, we present the first human specimen-based study using gene expression as a surrogate for miRNA activity patterns in sarcomas, while validating many of these miRNAs using a paraffin-embedded tissue cohort. Our analysis uncovers possible miRNA sequence alterations as a potential reason for differential activity, and we identify an association between RAS signaling and miRNA processing in which miRNAs may attenuate their own biogenesis. We show how relationships between miRNA activity and critical pathways can be assessed by high throughput genome-wide analysis. The logical next step would be a “Systems” level integration of miRNA, mRNA, and proteomic data, which would allow more comprehensive and definitive explorations of the role of miRNAs in mesenchymal tumors, and other malignancies.

## Competing interests

The authors declare that they have no competing interests.

## Authors’ contributions

EF, ADK, JQ, and DS conceived the study. EF, ADK, NF, MC, LH, RR, and DS conducted experiments and performed data analysis. EF, ADK, ARP, JG, PAK, MCG, JQ, and DS wrote the manuscript. All authors read and approved the final manuscript.

## Financial support

Supported by the National Institutes of Health [U19 CA148065 to J.Q., K22 CA138716-01A2 to D.S.]; the Dana-Farber Cancer Institute Strategic Plan Fund [to M.C., J.Q.]; and a donation by Dr Richard and Virginia Clemmer to the sarcoma program at Beth Israel Deaconess Medical Center [to D.S., M.C.G.].

## Supplementary Material

Additional file 1 **Table S1.** Commonly activated and silenced miRNAs across all sarcoma histological subtypes. A superscript 1 denotes miRNAs with potential sequence alterations. **Table S2.** miRNA activity patterns in osteosarcoma. Osteosarcoma-specific deregulation patterns compared to all normal tissue (p = 0.005 and FDR = 0.01). **Table S3.** miRNA activity patterns in osteosarcoma. Osteosarcoma-specific deregulation patterns compared to mesenchymal normal tissue (p = 0.005 and FDR = 0.01). **Table S4.** miRNAs commonly activated or silenced in sarcomas and epithelial cancers. **Table S5.** miRNAs deregulated in the “RAS active” group (p = 0.005 and FDR = 0.01). **Table S6.** miRNAs deregulated in the “RAS non-active” group (p = 0.005 and FDR = 0.01). **Table S7.** RAS-switching miRNAs by sarcoma histological subtype. Boldface denotes miRNAs which overlap with those identified in the aggregate RAS pathway analysis. **Table S8.** Biological themes represented in distinct miRNA activity patterns. Boldface denotes the pathways discussed in the main text of the manuscript. Click here for file
