# Supplementary material for: A microRNA activity map of human mesenchymal tumors: connections to oncogenic pathways; an integrative transcriptomic study
Source: BMC Genomics. 2012 Jul 23;13:332. doi: 10.1186/1471-2164-13-332 (PMC3443663; doi:10.1186/1471-2164-13-332)
Supplement: Additional file 1 — Table S1. Commonly activated and silenced miRNAs across all sarcoma histological subtypes. A superscript 1 denotes miRNAs with potential sequence alterations. Table S2. miRNA activity patterns in osteosarcoma. Osteosarcoma-specific deregulation patterns compared to all normal tissue (p = 0.005 and FDR = 0.01). Table S3. miRNA activity patterns in osteosarcoma. Osteosarcoma-specific deregulation patterns compared to mesenchymal normal tissue (p = 0.005 and FDR = 0.01). Table S4. miRNAs commonly activated or silenced in sarcomas and epithelial cancers. Table S5. miRNAs deregulated in the “RAS active” group (p = 0.005 and FDR = 0.01). Table S6. miRNAs deregulated in the “RAS non-active” group (p = 0.005 and FDR = 0.01). Table S7. RAS-switching miRNAs by sarcoma histological subtype. Boldface denotes miRNAs which overlap with those identified in the aggregate RAS pathway analysis. Table S8. Biological themes represented in distinct miRNA activity patterns. Boldface denotes the pathways discussed in the main text of the manuscript. [file 1471-2164-13-332-S1.doc]

**Supplemental Information**

**Table S1. Commonly activated and silenced miRNAs across all sarcoma histological subtypes. A superscript 1 denotes miRNAs with potential sequence alterations.**

| **VS ALL NORMAL TISSUE SAMPLES** | | **VS MESENCHYMAL NORMAL TISSUE SAMPLES** | |
| --- | --- | --- | --- |
| **Activated miRNAs** | **Silenced miRNAs** | **Activated miRNAs** | **Silenced miRNAs** |
| hsa-miR-133b | hsa-miR-380-3p | hsa-miR-296 | hsa-miR-21 |
| hsa-miR-331 | hsa-miR-186¹ | hsa-miR-328¹ | hsa-miR-30e-3p |
| hsa-miR-320 | hsa-miR-33 | hsa-miR-331 | hsa-miR-30a-5p |
| hsa-let-7e¹ | hsa-miR-19b¹ | hsa-miR-326 | hsa-miR-106b |
| hsa-miR-24¹ | hsa-miR-448 | hsa-miR-149 | hsa-miR-9* |
| hsa-miR-185¹ | hsa-miR-302b* | hsa-miR-423 | hsa-miR-20 |
| hsa-miR-198 | hsa-miR-429 | hsa-miR-339 | hsa-miR-106a |
| hsa-let-7c¹ | hsa-miR-101¹ | hsa-miR-133b | hsa-miR-200c |
| hsa-miR-10b | hsa-miR-144 | hsa-miR-133a | hsa-miR-302b |
| hsa-miR-125a | hsa-miR-203¹ | hsa-miR-324-5p¹ | hsa-miR-139 |
| hsa-miR-99b | hsa-miR-137 | hsa-miR-370 | hsa-miR-33 |
| hsa-miR-133a | hsa-miR-200b¹ | hsa-miR-346 | hsa-miR-181a |
| hsa-miR-204 | hsa-miR-32¹ | hsa-miR-24¹ | hsa-miR-130a |
| hsa-miR-324-3p | hsa-miR-19a¹ | hsa-miR-378¹ | hsa-miR-335 |
| hsa-miR-28 | hsa-miR-26b¹ | hsa-miR-371 | hsa-miR-19a¹ |
| hsa-let-7i¹ | hsa-miR-367 | hsa-miR-125a | hsa-miR-448 |
| hsa-miR-365 | hsa-miR-301 | hsa-let-7b¹ | hsa-miR-19b¹ |
| hsa-miR-22¹ |  | hsa-miR-125b¹ | hsa-miR-186¹ |
| hsa-miR-125b¹ |  | hsa-miR-198 | hsa-miR-302c* |
| hsa-miR-370 |  | hsa-miR-127 | hsa-miR-217 |
| hsa-miR-378¹ |  | hsa-miR-193 | hsa-miR-30e-5p |
| hsa-miR-339 |  | hsa-miR-324-3p | hsa-miR-380-3p |
| hsa-miR-346 |  | hsa-miR-28 | hsa-miR-32¹ |
| hsa-miR-184 |  | hsa-miR-337 | hsa-miR-203¹ |
| hsa-let-7d¹ |  | hsa-let-7c¹ | hsa-miR-367 |
| hsa-miR-197¹ |  | hsa-miR-188 | hsa-miR-137 |
| hsa-miR-214¹ |  | hsa-miR-340¹ | hsa-miR-302b* |
| hsa-miR-345 |  | hsa-miR-214¹ | hsa-miR-301 |
| hsa-miR-150 |  | hsa-let-7e¹ | hsa-miR-26b¹ |
| hsa-miR-340¹ |  | hsa-miR-345 | hsa-miR-200b¹ |
| hsa-miR-34a¹ |  | hsa-miR-34a¹ | hsa-miR-1 |
| hsa-miR-422b |  | hsa-let-7d¹ | hsa-miR-155 |
| hsa-let-7b¹ |  | hsa-let-7i¹ | hsa-miR-429 |
| hsa-miR-145¹ |  | hsa-miR-184 | hsa-miR-101¹ |
| hsa-miR-324-5p¹ |  | hsa-miR-449 | hsa-miR-144 |
| hsa-miR-138 |  | hsa-miR-422a¹ |  |
| hsa-miR-296 |  | hsa-miR-150 |  |
| hsa-miR-326 |  | hsa-let-7a¹ |  |
| hsa-miR-328¹ |  | hsa-miR-197¹ |  |
| hsa-miR-34b |  | hsa-miR-320 |  |
| hsa-miR-34c |  | hsa-miR-138 |  |
| hsa-miR-371 |  | hsa-miR-425¹ |  |
| hsa-miR-193 |  | hsa-miR-185¹ |  |
| hsa-miR-210¹ |  | hsa-miR-187 |  |
| hsa-miR-187 |  | hsa-miR-422b |  |
| hsa-miR-188 |  | hsa-miR-34c |  |
| hsa-miR-199a |  | hsa-miR-199a |  |
| hsa-miR-205 |  | hsa-miR-210¹ |  |
| hsa-miR-449 |  | hsa-miR-211 |  |
| hsa-miR-425¹ |  | hsa-miR-145¹ |  |
| hsa-miR-149 |  | hsa-miR-204 |  |
| hsa-miR-134 |  | hsa-miR-22¹ |  |
| hsa-miR-422a¹ |  |  |  |
| hsa-miR-127 |  |  |  |
| hsa-miR-337 |  |  |  |
| hsa-miR-423 |  |  |  |
| hsa-miR-10a |  |  |  |
| hsa-let-7a¹ |  |  |  |
| hsa-miR-211 |  |  |  |

**Table S2. miRNA activity patterns in osteosarcoma. Osteosarcoma-specific deregulation patterns compared to all normal tissue (p=0.005 and FDR=0.01).**

| **miRNA** | **score** | **p-value** | **q-value** |
| --- | --- | --- | --- |
| hsa-let-7g | 6.4812 | 0.0000 | 0.0073 |
| hsa-miR-10a | 6.7255 | 0.0000 | 0.0046 |
| hsa-miR-199a | 6.7563 | 0.0020 | 0.0045 |
| hsa-miR-205 | 6.7847 | 0.0000 | 0.0043 |
| hsa-miR-18 | 6.9178 | 0.0020 | 0.0032 |
| hsa-miR-34b | 7.0460 | 0.0000 | 0.0027 |
| hsa-miR-365 | 7.2024 | 0.0010 | 0.0020 |
| hsa-miR-122a | 7.2118 | 0.0010 | 0.0020 |
| hsa-miR-204 | 7.4270 | 0.0000 | 0.0014 |
| hsa-miR-22 | 7.6245 | 0.0000 | 0.0007 |
| hsa-miR-145 | 7.6748 | 0.0000 | 0.0006 |
| hsa-miR-320 | 7.6782 | 0.0000 | 0.0007 |
| hsa-miR-197 | 7.8553 | 0.0000 | 0.0004 |
| hsa-let-7a | 8.0511 | 0.0000 | 0.0002 |
| hsa-miR-185 | 8.1793 | 0.0000 | 0.0002 |
| hsa-miR-147 | 8.8246 | 0.0000 | 0.0001 |
| hsa-miR-138 | 8.8441 | 0.0000 | 0.0001 |
| hsa-miR-340 | 9.0733 | 0.0000 | 0.0001 |
| hsa-miR-211 | 9.2591 | 0.0000 | 0.0001 |
| hsa-let-7i | 9.3889 | 0.0000 | 0.0000 |
| hsa-miR-34c | 9.4260 | 0.0000 | 0.0000 |
| hsa-let-7d | 9.4488 | 0.0000 | 0.0000 |
| hsa-miR-422b | 9.4608 | 0.0000 | 0.0000 |
| hsa-miR-422a | 9.4808 | 0.0000 | 0.0000 |
| hsa-miR-187 | 9.6866 | 0.0000 | 0.0000 |
| hsa-miR-198 | 10.1060 | 0.0000 | 0.0000 |
| hsa-miR-134 | 10.1739 | 0.0000 | 0.0000 |
| hsa-miR-150 | 10.2574 | 0.0000 | 0.0000 |
| hsa-let-7c | 10.4979 | 0.0000 | 0.0000 |
| hsa-miR-184 | 10.5984 | 0.0000 | 0.0000 |
| hsa-miR-345 | 10.6037 | 0.0000 | 0.0000 |
| hsa-miR-449 | 10.6190 | 0.0000 | 0.0000 |
| hsa-miR-210 | 10.7332 | 0.0000 | 0.0000 |
| hsa-let-7e | 10.8151 | 0.0000 | 0.0000 |
| hsa-miR-28 | 11.0308 | 0.0000 | 0.0000 |
| hsa-miR-425 | 11.0641 | 0.0000 | 0.0000 |
| hsa-miR-188 | 11.3684 | 0.0000 | 0.0000 |
| hsa-miR-193 | 11.4054 | 0.0000 | 0.0000 |
| hsa-miR-337 | 11.8074 | 0.0000 | 0.0000 |
| hsa-let-7b | 12.8713 | 0.0000 | 0.0000 |
| hsa-miR-125b | 12.8818 | 0.0000 | 0.0000 |
| hsa-miR-34a | 13.0433 | 0.0000 | 0.0000 |
| hsa-miR-378 | 13.1505 | 0.0000 | 0.0000 |
| hsa-miR-127 | 13.7552 | 0.0000 | 0.0000 |
| hsa-miR-324-3p | 13.8403 | 0.0000 | 0.0000 |
| hsa-miR-371 | 13.8974 | 0.0000 | 0.0000 |
| hsa-miR-24 | 13.9253 | 0.0000 | 0.0000 |
| hsa-miR-214 | 14.0645 | 0.0000 | 0.0000 |
| hsa-miR-125a | 14.4700 | 0.0000 | 0.0000 |
| hsa-miR-346 | 14.6648 | 0.0000 | 0.0000 |
| hsa-miR-324-5p | 14.6685 | 0.0000 | 0.0000 |
| hsa-miR-149 | 14.8075 | 0.0000 | 0.0000 |
| hsa-miR-370 | 15.4431 | 0.0000 | 0.0000 |
| hsa-miR-133a | 15.7334 | 0.0000 | 0.0000 |
| hsa-miR-133b | 15.8058 | 0.0000 | 0.0000 |
| hsa-miR-339 | 16.0081 | 0.0000 | 0.0000 |
| hsa-miR-423 | 16.8701 | 0.0000 | 0.0000 |
| hsa-miR-328 | 17.6962 | 0.0000 | 0.0000 |
| hsa-miR-331 | 18.3154 | 0.0000 | 0.0000 |
| hsa-miR-296 | 18.9506 | 0.0000 | 0.0000 |
| hsa-miR-326 | 19.0306 | 0.0000 | 0.0000 |
| hsa-miR-144 | -8.8688 | 0.0000 | 0.0034 |
| hsa-miR-19a | -8.5640 | 0.0000 | 0.0034 |

**Table S3. miRNA activity patterns in osteosarcoma. Osteosarcoma-specific deregulation patterns** compared to mesenchymal normal tissue (p=0.005 and FDR=0.01).

| **miRNA** | **score** | **p-value** | **q-value** |
| --- | --- | --- | --- |
| hsa-miR-331 | 15.2003 | 0 | 0 |
| hsa-miR-326 | 14.4583 | 0 | 0 |
| hsa-miR-296 | 14.3416 | 0 | 0 |
| hsa-miR-328 | 13.7816 | 0 | 0 |
| hsa-miR-339 | 13.2466 | 0 | 0 |
| hsa-miR-149 | 12.6832 | 0 | 0 |
| hsa-miR-423 | 12.5331 | 0 | 0 |
| hsa-miR-370 | 12.4985 | 0 | 0 |
| hsa-miR-127 | 12.2017 | 0 | 0 |
| hsa-miR-133b | 12.1896 | 0 | 0 |
| hsa-miR-324-5p | 12.0988 | 0 | 0 |
| hsa-miR-346 | 11.9081 | 0 | 0 |
| hsa-miR-133a | 11.9067 | 0 | 0 |
| hsa-miR-324-3p | 11.2615 | 0 | 0 |
| hsa-miR-371 | 11.0359 | 0 | 0 |
| hsa-miR-345 | 10.733 | 0 | 0 |
| hsa-miR-34a | 10.4599 | 0 | 0 |
| hsa-miR-24 | 10.3752 | 0 | 0 |
| hsa-miR-138 | 10.2791 | 0 | 0 |
| hsa-miR-125a | 9.91861 | 0 | 0 |
| hsa-miR-337 | 9.75728 | 0 | 0 |
| hsa-miR-193 | 9.56119 | 0 | 0 |
| hsa-miR-150 | 9.21789 | 0 | 0 |
| hsa-let-7b | 9.17401 | 0 | 0 |
| hsa-miR-378 | 9.11847 | 0 | 9.25325E-05 |
| hsa-let-7e | 9.10546 | 0 | 8.89735E-05 |
| hsa-miR-214 | 8.88173 | 0 | 8.56782E-05 |
| hsa-miR-184 | 8.8637 | 0 | 8.26183E-05 |
| hsa-let-7a | 8.78126 | 0 | 0.000119654 |
| hsa-miR-125b | 8.70582 | 0 | 0.000115666 |
| hsa-miR-188 | 8.6787 | 0 | 0.000111934 |
| hsa-miR-340 | 8.62708 | 0 | 0.000108437 |
| hsa-miR-211 | 8.35355 | 0 | 0.000315452 |
| hsa-let-7c | 8.33308 | 0 | 0.000306174 |
| hsa-let-7d | 8.21995 | 0 | 0.000396568 |
| hsa-miR-197 | 8.12587 | 0 | 0.000514069 |
| hsa-miR-425 | 8.11518 | 0.001 | 0.000500176 |
| hsa-miR-134 | 8.06812 | 0 | 0.00054789 |
| hsa-miR-449 | 8.06207 | 0 | 0.000533841 |
| hsa-miR-198 | 8.05773 | 0 | 0.000549412 |
| hsa-miR-34c | 8.02268 | 0 | 0.000536011 |
| hsa-miR-210 | 7.82477 | 0.001 | 0.000908801 |
| hsa-miR-147 | 7.73949 | 0 | 0.00104906 |
| hsa-miR-204 | 7.59701 | 0 | 0.00136696 |
| hsa-miR-422a | 7.16306 | 0.003 | 0.00344427 |
| hsa-let-7i | 7.07475 | 0 | 0.00407344 |
| hsa-miR-187 | 7.04222 | 0 | 0.00423287 |
| hsa-miR-422b | 6.99869 | 0.001 | 0.00443385 |
| hsa-miR-375 | 6.86622 | 0.002 | 0.00561804 |
| hsa-miR-122a | 6.73469 | 0.002 | 0.00735633 |
| hsa-miR-28 | 6.73236 | 0.002 | 0.00723477 |

**Table S4**. miRNAs commonly activated or silenced in sarcomas and epithelial cancers

| **Activated in all sarcomas** | **Activated in HNC and Ovarian samples** | **Overlap** |
| --- | --- | --- |
| hsa-miR-296 | hsa-let-7a | hsa-let-7a |
| hsa-miR-328 | hsa-let-7b | hsa-let-7b |
| hsa-miR-331 | hsa-let-7c | hsa-let-7c |
| hsa-miR-326 | hsa-let-7d | hsa-let-7d |
| hsa-miR-149 | hsa-let-7e | hsa-let-7e |
| hsa-miR-423 | hsa-let-7i | hsa-let-7i |
| hsa-miR-339 | hsa-miR-122a | hsa-miR-125a |
| hsa-miR-133b | hsa-miR-125a | hsa-miR-125b |
| hsa-miR-133a | hsa-miR-125b | hsa-miR-127 |
| hsa-miR-324-5p | hsa-miR-127 | hsa-miR-133a |
| hsa-miR-370 | hsa-miR-133a | hsa-miR-133b |
| hsa-miR-346 | hsa-miR-133b | hsa-miR-138 |
| hsa-miR-24 | hsa-miR-134 | hsa-miR-149 |
| hsa-miR-378 | hsa-miR-138 | hsa-miR-150 |
| hsa-miR-371 | hsa-miR-147 | hsa-miR-184 |
| hsa-miR-125a | hsa-miR-149 | hsa-miR-185 |
| hsa-let-7b | hsa-miR-150 | hsa-miR-187 |
| hsa-miR-125b | hsa-miR-184 | hsa-miR-188 |
| hsa-miR-198 | hsa-miR-185 | hsa-miR-193 |
| hsa-miR-127 | hsa-miR-187 | hsa-miR-197 |
| hsa-miR-193 | hsa-miR-188 | hsa-miR-198 |
| hsa-miR-324-3p | hsa-miR-193 | hsa-miR-204 |
| hsa-miR-28 | hsa-miR-197 | hsa-miR-210 |
| hsa-miR-337 | hsa-miR-198 | hsa-miR-211 |
| hsa-let-7c | hsa-miR-204 | hsa-miR-214 |
| hsa-miR-188 | hsa-miR-210 | hsa-miR-22 |
| hsa-miR-340 | hsa-miR-211 | hsa-miR-24 |
| hsa-miR-214 | hsa-miR-214 | hsa-miR-28 |
| hsa-let-7e | hsa-miR-22 | hsa-miR-296 |
| hsa-miR-345 | hsa-miR-24 | hsa-miR-320 |
| hsa-miR-34a | hsa-miR-28 | hsa-miR-324-3p |
| hsa-let-7d | hsa-miR-296 | hsa-miR-324-5p |
| hsa-let-7i | hsa-miR-320 | hsa-miR-326 |
| hsa-miR-184 | hsa-miR-324-3p | hsa-miR-328 |
| hsa-miR-449 | hsa-miR-324-5p | hsa-miR-331 |
| hsa-miR-422a | hsa-miR-326 | hsa-miR-337 |
| hsa-miR-150 | hsa-miR-328 | hsa-miR-339 |
| hsa-let-7a | hsa-miR-331 | hsa-miR-340 |
| hsa-miR-197 | hsa-miR-337 | hsa-miR-345 |
| hsa-miR-320 | hsa-miR-339 | hsa-miR-346 |
| hsa-miR-138 | hsa-miR-340 | hsa-miR-34a |
| hsa-miR-425 | hsa-miR-345 | hsa-miR-34c |
| hsa-miR-185 | hsa-miR-346 | hsa-miR-370 |
| hsa-miR-187 | hsa-miR-34a | hsa-miR-371 |
| hsa-miR-422b | hsa-miR-34c | hsa-miR-378 |
| hsa-miR-34c | hsa-miR-370 | hsa-miR-422a |
| hsa-miR-199a | hsa-miR-371 | hsa-miR-422b |
| hsa-miR-210 | hsa-miR-378 | hsa-miR-423 |
| hsa-miR-211 | hsa-miR-422a | hsa-miR-425 |
| hsa-miR-145 | hsa-miR-422b | hsa-miR-449 |
| hsa-miR-204 | hsa-miR-423 |  |
| hsa-miR-22 | hsa-miR-425 |  |
|  | hsa-miR-449 |  |
| **Silenced in all sarcomas** | **Silenced in HNC and Ovarian samples** | **Overlap** |
| hsa-miR-380-3p | hsa-miR-144 | hsa-miR-144 |
| hsa-miR-186 |  |  |
| hsa-miR-33 |  |  |
| hsa-miR-19b |  |  |
| hsa-miR-448 |  |  |
| hsa-miR-302b* |  |  |
| hsa-miR-429 |  |  |
| hsa-miR-101 |  |  |
| hsa-miR-144 |  |  |
| hsa-miR-203 |  |  |
| hsa-miR-137 |  |  |
| hsa-miR-200b |  |  |
| hsa-miR-32 |  |  |
| hsa-miR-19a |  |  |
| hsa-miR-26b |  |  |
| hsa-miR-367 |  |  |
| hsa-miR-301 |  |  |

**Table S5. miRNAs deregulated in the “RAS active” group (p=0.005 and FDR=0.01).**

| **miRNA** | **score** | **p-value** | **q-value** |
| --- | --- | --- | --- |
| hsa-miR-330 | 9.4976 | 0.0000 | 0.0000 |
| hsa-miR-342 | 9.6422 | 0.0000 | 0.0000 |
| hsa-miR-200a | 9.2841 | 0.0000 | 0.0007 |
| hsa-miR-141 | 8.7841 | 0.0000 | 0.0039 |
| hsa-miR-15a | 7.8089 | 0.0000 | 0.0042 |
| hsa-miR-214 | 7.8491 | 0.0000 | 0.0042 |
| hsa-miR-27a | 7.8124 | 0.0000 | 0.0044 |
| hsa-miR-99b | 7.8662 | 0.0010 | 0.0045 |
| hsa-miR-31 | 8.3347 | 0.0000 | 0.0046 |
| hsa-miR-211 | 7.7187 | 0.0000 | 0.0046 |
| hsa-miR-134 | 7.8766 | 0.0000 | 0.0047 |
| hsa-miR-324-3p | 8.4301 | 0.0000 | 0.0047 |
| hsa-miR-424 | 7.8880 | 0.0000 | 0.0049 |
| hsa-miR-10a | 7.5721 | 0.0000 | 0.0053 |
| hsa-miR-15b | 7.5314 | 0.0000 | 0.0054 |
| hsa-miR-99a | 7.9014 | 0.0000 | 0.0054 |
| hsa-miR-200b | 7.5545 | 0.0010 | 0.0054 |
| hsa-miR-204 | 7.6020 | 0.0000 | 0.0054 |
| hsa-miR-217 | 7.5354 | 0.0000 | 0.0054 |
| hsa-miR-128b | 7.5874 | 0.0000 | 0.0054 |
| hsa-miR-367 | 8.1229 | 0.0000 | 0.0054 |
| hsa-miR-220 | 8.0413 | 0.0000 | 0.0055 |
| hsa-miR-129 | 7.5432 | 0.0000 | 0.0055 |
| hsa-miR-27b | 7.9416 | 0.0000 | 0.0055 |
| hsa-miR-22 | 7.4599 | 0.0000 | 0.0056 |
| hsa-miR-195 | 7.4401 | 0.0000 | 0.0056 |
| hsa-miR-200c | 7.4188 | 0.0000 | 0.0056 |
| hsa-miR-7 | 7.4968 | 0.0000 | 0.0057 |
| hsa-miR-140 | 7.3941 | 0.0000 | 0.0058 |
| hsa-miR-10b | 7.3515 | 0.0000 | 0.0059 |
| hsa-miR-16 | 8.1465 | 0.0000 | 0.0062 |
| hsa-miR-100 | 7.2953 | 0.0010 | 0.0065 |
| hsa-miR-125b | 7.1975 | 0.0000 | 0.0074 |
| hsa-miR-213 | 7.1685 | 0.0000 | 0.0075 |
| hsa-miR-194 | 7.1742 | 0.0000 | 0.0076 |
| hsa-miR-146 | 7.1187 | 0.0010 | 0.0081 |
| hsa-miR-128a | 7.1255 | 0.0000 | 0.0082 |
| hsa-miR-149 | 7.0376 | 0.0020 | 0.0087 |
| hsa-miR-210 | 7.0573 | 0.0000 | 0.0089 |
| hsa-miR-193 | 7.0383 | 0.0010 | 0.0089 |
| hsa-miR-9* | 7.0099 | 0.0000 | 0.0091 |
| hsa-miR-29a | 6.9591 | 0.0000 | 0.0099 |

**Table S6. miRNAs deregulated in the “RAS non-active” group (p=0.005 and FDR=0.01).**

| **miRNA** | **score** | **p-value** | **q-value** |
| --- | --- | --- | --- |
| hsa-miR-141 | -9.3610 | 0.0000 | 0.0000 |
| hsa-miR-200a | -10.2846 | 0.0000 | 0.0000 |
| hsa-miR-330 | -10.3464 | 0.0000 | 0.0000 |
| hsa-miR-367 | -9.5400 | 0.0000 | 0.0000 |
| hsa-miR-200b | -9.1068 | 0.0000 | 0.0008 |
| hsa-miR-217 | -8.9802 | 0.0000 | 0.0010 |
| hsa-miR-200c | -8.5809 | 0.0000 | 0.0014 |
| hsa-miR-27b | -8.4418 | 0.0000 | 0.0015 |
| hsa-miR-424 | -8.3218 | 0.0000 | 0.0022 |
| hsa-miR-99a | -8.1558 | 0.0000 | 0.0031 |
| hsa-miR-9* | -8.0162 | 0.0020 | 0.0037 |
| hsa-miR-448 | -7.8373 | 0.0000 | 0.0050 |
| hsa-miR-342 | -7.8128 | 0.0000 | 0.0051 |
| hsa-miR-7 | -7.6461 | 0.0000 | 0.0053 |
| hsa-miR-15a | -7.7505 | 0.0000 | 0.0054 |
| hsa-miR-142-3p | -7.6747 | 0.0000 | 0.0054 |
| hsa-miR-181a | -7.4336 | 0.0000 | 0.0063 |
| hsa-miR-369 | -7.3246 | 0.0000 | 0.0065 |
| hsa-miR-101 | -7.4425 | 0.0000 | 0.0065 |
| hsa-miR-19b | -7.5134 | 0.0000 | 0.0065 |
| hsa-miR-151 | -7.3595 | 0.0000 | 0.0066 |
| hsa-miR-429 | -7.3281 | 0.0000 | 0.0067 |
| hsa-miR-31 | -7.4717 | 0.0010 | 0.0067 |
| hsa-miR-194 | -7.3682 | 0.0000 | 0.0068 |
| hsa-miR-27a | -7.4430 | 0.0000 | 0.0069 |
| hsa-miR-146 | -7.3746 | 0.0000 | 0.0071 |
| hsa-miR-323 | -7.2201 | 0.0000 | 0.0080 |
| hsa-miR-134 | -7.2055 | 0.0000 | 0.0080 |
| hsa-miR-16 | -7.1269 | 0.0000 | 0.0096 |
| hsa-miR-220 | -7.0980 | 0.0000 | 0.0096 |

**Table S7. RAS-switching miRNAs by sarcoma histological subtype.** Boldface denotes miRNAs which overlap with those identified in the aggregate RAS pathway analysis.

| **RAS-switching miRNAs** | **Non-myxoid Lipo** | **Synovial** |
| --- | --- | --- |
|  |  |  |
|  | **hsa-miR-200a** | **hsa-miR-330** |
|  | **hsa-miR-141** | **hsa-miR-367** |
|  | **hsa-miR-367** | **hsa-miR-342** |
|  | **hsa-miR-200b** | **hsa-miR-200b** |
|  | **hsa-miR-424** | **hsa-miR-217** |
|  | **hsa-miR-200c** | **hsa-miR-424** |
|  | **hsa-miR-31** | **hsa-miR-99a** |
|  | **hsa-miR-146** | **hsa-miR-200c** |
|  | hsa-miR-144 | **hsa-miR-31** |
|  | hsa-miR-302b* | **hsa-miR-15a** |
|  | hsa-miR-302a* | **hsa-miR-7** |
|  |  | **hsa-miR-96** |
|  |  | **hsa-miR-194** |
|  |  | **hsa-miR-146** |
|  |  | hsa-miR-144 |
|  |  | hsa-miR-26b |
|  |  | hsa-miR-33 |
|  |  | hsa-miR-335 |
|  |  | hsa-miR-137 |
|  |  | hsa-miR-106b |
|  |  | hsa-miR-199a* |

**Table S8. Biological themes represented in distinct miRNA activity patterns.**  Boldface denotes the pathways discussed in the main text of the manuscript.

| **SUBTYPE** | **BIOLOGICAL THEMES-PATHWAYS** | **EASE scores** | **miRNA** |
| --- | --- | --- | --- |
| LEIO | | Transferase | | --- | | Serine/threonine-specific phosphatase | | [Serine/threonine specific protein phosphatases signature](http://us.expasy.org/cgi-bin/prosite-search-ac?PDOC00115) | | [Chondroitin /Heparan sulfate biosynthesis - Homo sapiens](http://www.genome.ad.jp/dbget-bin/show_pathway?hsa00532) | | Growth factor receptor | | [Tyrosine protein kinases specific active-site signature](http://us.expasy.org/cgi-bin/prosite-search-ac?PDOC00100) | | Phosphotransferase | | | 0.00 | | --- | | 0.02 | | 0.02 | | 0.02 | | 0.03 | | 0.03 | | 0.04 | | hsa-miR-100 |
|  | \| Transferase \| \| --- \| \| Serine/threonine-specific phosphatase \| \| [Serine/threonine specific protein phosphatases signature](http://us.expasy.org/cgi-bin/prosite-search-ac?PDOC00115) \| \| [Chondroitin /Heparan sulfate biosynthesis - Homo sapiens](http://www.genome.ad.jp/dbget-bin/show_pathway?hsa00532) \| \| Growth factor receptor \| \| [Tyrosine protein kinases specific active-site signature](http://us.expasy.org/cgi-bin/prosite-search-ac?PDOC00100) \| \| Phosphotransferase \| | \| 0.005 \| \| --- \| \| 0.02 \| \| 0.02 \| \| 0.02 \| \| 0.03 \| \| 0.03 \| \| 0.04 \| | hsa-miR-99a |
| LIPO | | DNA-binding protein | | --- | | Pol II transcription | | DNA binding | | serine/threonine-specific protein kinase | | Protein kinases ATP-binding region signature | | Phosphotransferase | | Transcription factor | | Serine/Threonine protein kinases active-site signature | | Cell cycle - Homo sapiens | | Nuclear hormones receptors DNA-binding region signature | | Ribosomal protein S6 kinase II | | Hs_Nuclear Receptors | | Hs_Cell cycle | | Cell Growth and Death - Homo sapiens | | ATP | | | 0.000 | | --- | | 0.000 | | 0.000 | | 0.002 | | 0.002 | | 0.003 | | 0.005 | | 0.005 | | 0.005 | | 0.006 | | 0.006 | | 0.008 | | 0.009 | | 0.010 | | 0.011 | | hsa-miR-302a* |
|  | | **Extracellular matrix** | | --- | | Hydroxylysine | | Trimer | | Triple helix | | Hydroxyproline | | Coiled coil | | Basement membrane | | [Collagen alpha 1(IV) chain superfamily](http://pir.georgetown.edu/cgi-bin/pirwww/nbrfget?uid=SA1743&db=A) | | [Collagen alpha 1(IV) chain](http://pir.georgetown.edu/cgi-bin/ipcSF?id=SF002258) | | DNA-binding protein | | [Neurodegenerative Disorders - Homo sapiens](http://www.genome.ad.jp/dbget-bin/show_pathway?hsa01510) | | [**Hs_Inflammatory Response Pathway**](http://www.genmapp.org/MAPPSet-Human/GenMAPP.org_MAPPs/Other_MAPPs/Hs_Inflammatory_Response_Pathway.htm) | | [Fibrillar collagen carboxyl-terminal homology](http://pir.georgetown.edu/cgi-bin/pirwww/nbrfget?uid=DA1608&db=A) | | [Human retinoblastoma binding protein 2](http://pir.georgetown.edu/cgi-bin/ipcSF?id=SF002394) | | Cell binding | | Pol II transcription | | | 0.000 | | --- | | 0.000 | | 0.000 | | 0.000 | | 0.000 | | 0.000 | | 0.000 | | 0.000 | | 0.000 | | 0.002 | | 0.002 | | 0.003 | | 0.004 | | 0.004 | | 0.004 | | 0.004 | | hsa-miR-29c |
|  | \| Activator \| \| --- \| \| Pol II transcription \| \| DNA binding \| \| [Human transcription factor 3](http://pir.georgetown.edu/cgi-bin/pirwww/nbrfget?uid=FA1502&db=A) \| \| [Human transcription factor 3](http://pir.georgetown.edu/cgi-bin/ipcSF?id=SF001708) \| \| Proto-oncogene \| \| Myristylation \| \| Transcription factor \| \| [Cell Growth and Death - Homo sapiens](http://www.genome.ad.jp/dbget-bin/show_pathway?hsa01420) \| \| DNA-binding protein \| \| Transcription regulation \| \| [Cell cycle - Homo sapiens](http://www.genome.ad.jp/dbget-bin/show_pathway?hsa04110) \| \| Nuclear-cytoplasmic transport \| | \| 0.000 \| \| --- \| \| 0.000 \| \| 0.001 \| \| 0.001 \| \| 0.001 \| \| 0.001 \| \| 0.003 \| \| 0.003 \| \| 0.005 \| \| 0.008 \| \| 0.011 \| \| 0.012 \| \| 0.016 \| | hsa-miR-221 |
